# Utility of Whole Genome Sequencing for Population Screening of Deafness-Related Genetic Variants and Cytomegalovirus Infection in Newborns

**DOI:** 10.3389/fgene.2022.883617

**Published:** 2022-04-29

**Authors:** Jiale Xiang, Hongfu Zhang, Xiangzhong Sun, Junqing Zhang, Zhenpeng Xu, Jun Sun, Zhiyu Peng

**Affiliations:** ^1^ College of Life Sciences, University of Chinese Academy of Sciences, Beijing, China; ^2^ BGI Genomics, BGI-Shenzhen, Shenzhen, China; ^3^ Tianjin Medical Laboratory, BGI-Tianjin, BGI-Shenzhen, Tianjin, China; ^4^ BGI-Wuhan Clinical Laboratories, BGI-Shenzhen, Wuhan, China

**Keywords:** WGS, hearing loss, newborns, genetic variation, cytomegalovirus infection

## Abstract

**Background:** Hearing loss affects approximately two out of every 1,000 newborns. Genetic factors and congenital cytomegalovirus (CMV) infections account for around 90% of the etiology. The purpose of this study was to develop and test a whole genome sequencing (WGS) approach to detect deafness-related genetic variants and CMV infections simultaneously in newborns.

**Method:** Deafness-related genes causing congenital or childhood hearing loss were curated and selected for newborn screening. Nine dried blood spots from newborns with known genetic variants (n = 6) or CMV infections (n = 3) were employed to develop and validate the WGS testing and analytic pipeline. We then pilot tested the WGS analysis on 51 de-identified clinical samples.

**Results:** 92 gene-disease pairs were selected for screening hearing loss in newborns. In the validation test, WGS accurately detected all types of genetic variants, including single nucleotide variations, insertions/deletions, and copy number variations in the nuclear or mitochondrial genome. Sequence reads mapping to the CMV reference genome were discovered in CMV infected samples. In the pilot test, WGS identified nine out of 51 (18%) newborns carrying pathogenic variants associated with deafness.

**Conclusion:** WGS can simultaneously detect genetic variants and CMV infections in dried blood spot specimens from newborns. Our study provides proof of principle that genome sequencing can be a promising alternative for newborn screening of hearing loss.

## Introduction

Hearing loss has an estimated incidence of 0.1–0.3% in newborns, and its causes vary (Korver et al., 2017). Genetic factors are regarded as the primary contributors, accounting for 68% of the causes (Morton and Nance, 2006). Among the genetic factors, variants in both the nuclear and the mitochondrial genome can cause hereditary hearing loss. To date, over 150 genes have been linked with hearing loss in human, and novel associations are still being discovered every year (Azaiez et al., 2018).

Universal hearing screening in newborns has been implemented since the 1990s and has proven remarkably successful for the early identification of neonates with hearing loss, leading to interventions that have improved language development and social interaction (White et al., 2010). However, conventional newborns hearing screening (NHS) is incapable of identifying mild, delayed-onset/progressive or aminoglycoside-induced hearing loss. More importantly, it is unable to uncover the etiology of deafness (Kennedy et al., 2005).

Environmental factors are also important contributors. Congenital cytomegalovirus (CMV) infection is the most prevalent one, accounting for 21% of the prelingual hearing loss (Morton and Nance, 2006). With the advance of molecular technologies, screens for CMV or genetic variants at birth were conducted (Wang et al., 2019; Yamamoto et al., 2019). Newborn CMV screens use a polymerase chain reaction assay that can identify congenitally infected newborns, detecting those who pass auditory screens but would later develop hearing loss (Fowler et al., 2017; Yamamoto et al., 2019). Limited genetic screens using MALDI-TOF mass spectrometry or DNA microarray platforms were clinically adopted in China and were proven to compensate for the limitations of auditory screens by elucidating genetic etiologies and detecting infants who harbored mitochondrial variants and were at risk of aminoglycoside-induced ototoxicity (Dai et al., 2019; Wang et al., 2019; Guo et al., 2020). The feasibility of concurrent genetic and CMV screening in newborns was recently evaluated, and it was shown that the integration of these screening methods could identify the neonates with hearing loss missed by NHS (Lu et al., 2018).

Whole genome sequencing (WGS) is an attractive approach for NHS (Shearer et al., 2019). WGS can detect a wide range of types of genomic variation, including single nucleotide variations (SNV), small insertions and deletions (INDEL), and copy number variations (CNV) (Lindstrand et al., 2019). These variant types are common in genetic hearing loss (Shearer et al., 2014; Sloan-Heggen et al., 2016). In addition, WGS can detect mitochondrial variants which are associated with a predisposition to aminoglycoside ototoxicity and/or late-onset hearing loss (Usami and Nishio, 2004). Moreover, since the viral DNA of CMV is co-isolated with human genomic DNA from blood, WGS could theoretically detect CMV in dried blood specimens (Shearer et al., 2019). However, this approach has not yet been comprehensively evaluated.

In this study, we developed and validated an analytic pipeline using WGS to identify genetic variants and human CMV infections. We then pilot tested this pipeline on 51 de-identified clinical samples. Our study provides proof of principle that genome sequencing can be a promising alternative for newborn screening of hearing loss.

## Materials and Methods

### Study Design

This study was performed with the approval of the Institutional Review Board of BGI (IRB-20016-T2).

Deafness-related genes were curated and selected for WGS analysis in newborn screening. Nine dried blood spot samples were used to develop and validate the WGS analytic pipeline. These six positive samples with known deafness-related genetic variants were specifically selected, including compound heterozygous variants in *SLC26A4* (n = 1), compound heterozygous variants in *GJB2* (n = 1), homozygous variants in *MYO7A* (n = 1), a 3.7 Mb CNV (n = 1), and mitochondrial variants in *MT*-*RNR1* (n = 2). The selected samples covered all the variant types associated with hearing loss, including single nucleotide variations, insertions/deletions, and copy number variations, in heterozygous or homozygous statue. Homoplasmy and heteroplasmy of the mitochondrial variants were also covered. The three positive samples with CMV infections were specifically selected with a different level of viral loads. Following this validation analysis, we pilot tested the validated pipeline on samples from 51 newborns. All the dried blood spot samples were collected by heel prick form 3-day-old babies and routinely stored at room temperature.

### Gene Curation and Selection

A total of 174 gene-disease pairs and their associations were retrieved from clinical testing panels in the Genetic Testing Registry that had been curated by the ClinGen Hearing Loss Working Group (DiStefano et al., 2020). The exclusion criteria are: 1) the gene-disease association is lower than “strong” (Ceyhan-Birsoy et al., 2017); 2) hearing loss is not a presenting feature of the disease (DiStefano et al., 2019); or 3) the age of onset is greater than 18 years (Ceyhan-Birsoy et al., 2017). The youngest age at which individuals with pathogenic variants in a gene presented with disease was curated from the OMIM database. If the information was not available in the OMIM database, we integrated age of onset from the literature. Finally, two mitochondrial genes (*MT-RNR1* and *MT-TS1*) were added because *MT-RNR1* is definitively associated with maternally-inherited, aminoglycoside-induced hearing loss and *MT-TS1* is definitively associated with mitochondrial nonsyndromic hearing loss (DiStefano et al., 2019). Newborns who harbor mitochondrial variants generally pass the auditory screens because they are asymptomatic before they receive external stimulation such as aminoglycoside drugs (Wang et al., 2019).

### Sample Processing and WGS

DNA was extracted from dried blood spots using a QIAamp DNA Mini Kit (QIAGEN, Germany). Briefly, two dried blood spots were punched into a 1.5 ml Eppendorf tube with 360 μl Buffer ATL and incubated at 85°C for 10 min. A total of 40 μl proteinase K was added with incubation at 56°C for 60 min, followed by addition of 400 μl Buffer AL with incubation at 70°C for 10 min. Then, 400 μl ethanol was added and the sample was mixed by pulse-vortexing. The mixture was then transferred to a QIAamp Mini spin column and centrifuged at 6,000 × g for 1 min. 500 μl Buffer AW1 was added, followed by centrifuging at 8,000 × g for 1 min, and then 500 μl Buffer AW2 was added and the samples were centrifuged at 20,000 × g for 3 min. The QIAamp Mini spin column was placed in a clean 1.5 ml microcentrifuge tube with 80 µl Buffer TE and incubated at room temperature for 1 min. Finally, the tube was centrifuged at 12,000 × g for 1 min to collect the DNA.

**FIGURE 1:**
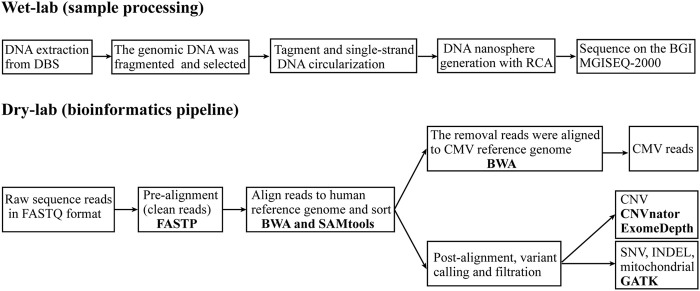
Overview of the whole genome sequencing and analytics pipeline. The WGS pipeline includes two sections: wet-lab (sample processing), which starts with DNA extraction from dried blood spots (DBS) and continues to generate a whole genome library that is sequenced on an MGISEQ-2000 sequencer; and dry-lab (bioinformatics pipeline), which starts with raw sequence reads in FASTQ format and continues with analysis using a series of software tools to identify genetic variants and reads from CMV. RCA, rolling circle amplification; BWA, Burrows-Wheeler Aligner; GATK, Genome Analysis Toolkit; CNV, copy number variant; SNV, single nucleotide variant; INDEL, small insertions and deletion; CMV, cytomegalovirus.

The genomic DNA was fragmented using covaris S220 (Covaris, Brighton, UK). DNA fragments between 100 bp and 300 bp were selected using AMPure XP beads (AGENCOURT). The selected DNA fragments were then repaired to obtain blunt ends and modified at the 3’ end to add dATP as a sticky end. A dTTP-tailed adapter sequence was ligated to both ends of the DNA fragments. The ligation product was heat-denatured together with splint oligos that were reverse-complementary to a specific strand of the product, and the single-strand molecule was ligated using DNA ligase. The remaining linear molecule was digested with exonuclease, obtaining a single-strand circular DNA library. The library was subjected to rolling circle amplification to generate DNA nanospheres. Whole-genome sequencing was then performed on an MGISEQ-2000 with 100-bp paired-end fragments (Figure 1).

### Bioinformatics Analysis

The WGS data were analyzed using our in-house WGS analysis pipeline following Genome Analysis Toolkit (GATK) best practices (DePristo et al., 2011). Briefly, clean reads were generated from the raw sequence reads in FASTQ format after quality control with FASTP (Chen et al., 2018). These were aligned to the human nuclear reference genome (GRCh37/hg19) and mitochondrial reference genome (GenBank:NC_012920) with the Burrows-Wheeler Aligner (BWA) (Li and Durbin, 2009) and then sorted with SAMtools to create binary alignment map (BAM) files (Li et al., 2009). Unmatched reads were mapped to the cytomegalovirus reference genome (NCBI Accession NC_006273.2) with BWA. CNV were analyzed using CNVnator (Abyzov et al., 2011) and ExomeDepth (Plagnol et al., 2012). The GATK was used to call SNV, INDEL and mitochondrial variants (Van der Auwera et al., 2013). The GATK Depth of Coverage tool was employed to obtain coverage statistics of genomic positions. The “well-coverage” category was defined as 20x coverage for nuclear variants (Sheppard et al., 2018) and 2000x coverage for mitochondrial variants (Schon et al., 2020). Using the ANNOVAR bioinformatics toolset to functionally annotate the genetic variants (Yang and Wang, 2015). Sequence variants were interpreted based on the expert specifications of variant interpretation guidelines for genetic hearing loss (Oza et al., 2018).

## Results

### Gene Selection for Newborn Hearing Screening

A total of 174 gene-disease pairs were retrieved from the ClinGen Hearing Loss Expert Panel. Of these, 74 were excluded because their associations were lower than strong. Eight gene-disease pairs were excluded because hearing loss is not a presenting feature of the disease: namely *ALMS1*/alstrom syndrome, *ATP6V1B1*/DRTA2, *BTD*/biotinidase deficiency, *COL4A5*/alport syndrome, *GJB3*/erythrokeratodermia variabilis, *DSPP*/dentinogenesis imperfecta, *GJB6*/clouston syndrome, and *TCOF1*/treacher-collins syndrome. Two gene-disease pairs (*COCH*/DFNA9 and *DNMT1*/ADCADN) characterized by progressive sensorineural hearing loss were excluded because of the disease’s age of onset. The age of onset for DFNA9 is approximate 20 years old (Robertson et al., 2006). The age of onset for ADCADN is usually from thirties to forties (Winkelmann et al., 2012). Two mitochondrial genes (*MT-RNR1* and *MT-TS1*) were manually added. *MT-RNR1* is definitively associated with maternally inherited, aminoglycoside-induced hearing loss, and *MT-TS1* is definitively associated with mitochondrial nonsyndromic hearing loss. Both gene-disease pairs were previously approved by the ClinGen Hearing Loss Expert Panel but were not listed on the ClinGen website (DiStefano et al., 2019).

Based on this, a total of 84 genes (92 gene-disease pairs) were finally included in the WGS analytic pipeline for screening hearing loss in newborns. The 84 genes included some of the genes most strongly linked with deafness and recommended for newborn screening, namely *GJB2*, *STRC*, *SLC26A4*, *MYO7A*, *TECTA*, *MYO15A*, *CDH23*, *USH2A*, *ADGRV1*, *WFS1, TMPRSS3, OTOF,* and *TMC1* (Shearer et al., 2019).

**FIGURE 2:**
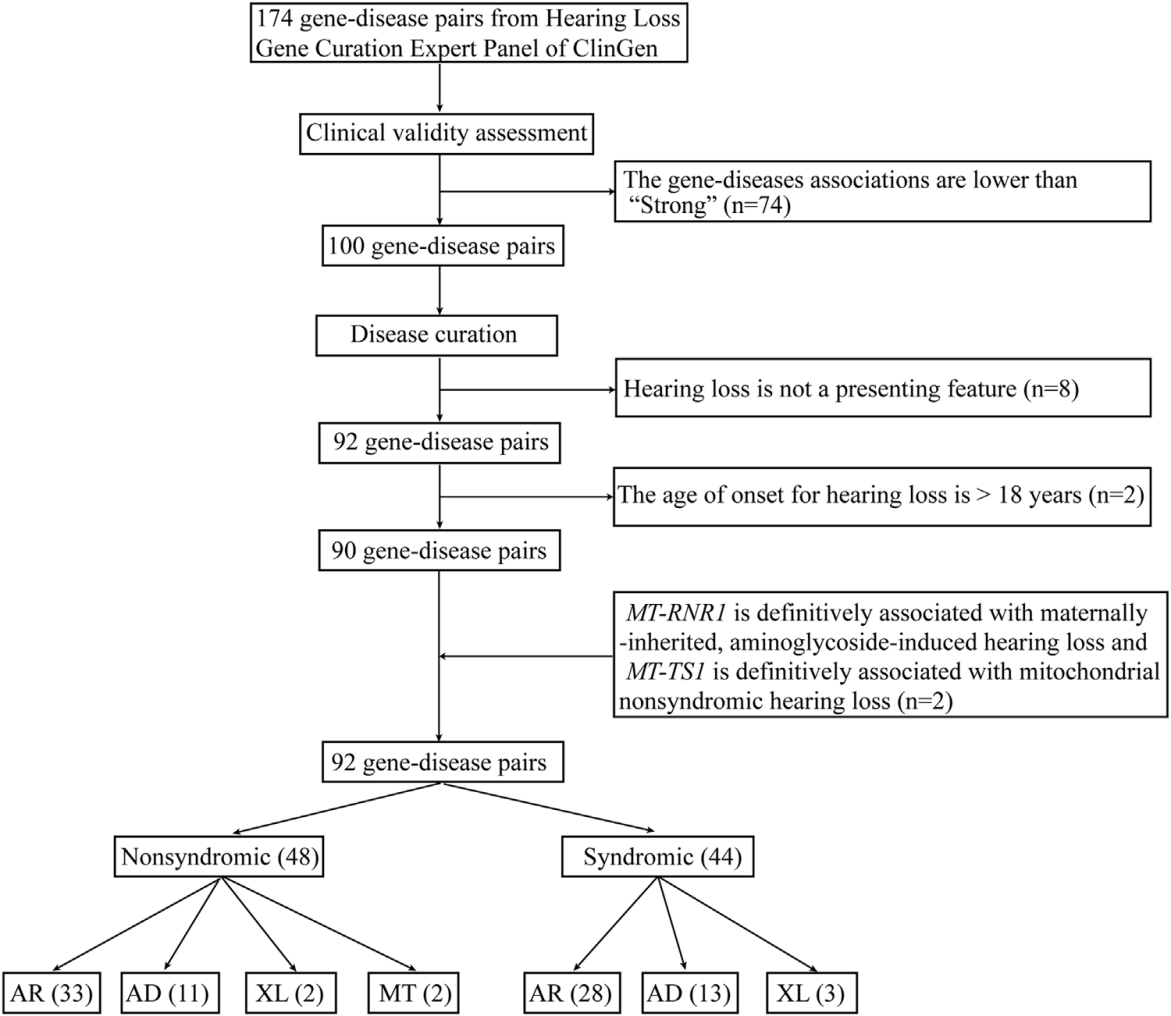
The workflow of gene curation for hearing loss. AR, autosomal recessive; AD, autosomal dominant; XL, X-linked; MT, mitochondrial.

Of these, 48 gene-disease pairs were associated with nonsyndromic hearing loss and 44 with syndromic hearing loss (Figure 2 and Supplementary Table S1). Autosomal recessive, autosomal dominant, X-linked and mitochondrial inheritance patterns accounted for 61 (66%), 24 (26%), 5 (6%), and 2 (2%) of the diseases, respectively.

### Validation of WGS Analytic Pipeline

To develop and validate a WGS pipeline for detecting genetic variants and CMV infections, WGS data from nine samples with known causes of deafness were analyzed. Overall, our sequencing produced a median of 99.54% mappable bases with coverage of at least 20X in the human nuclear genome and 98.92% mappable bases with coverage of at least 2000X in the mitochondrial genome (Supplementary Table S2). These metrics demonstrated that WGS data was of high quality and could be used to detect both nuclear and mitochondrial variants.

**FIGURE 3:**
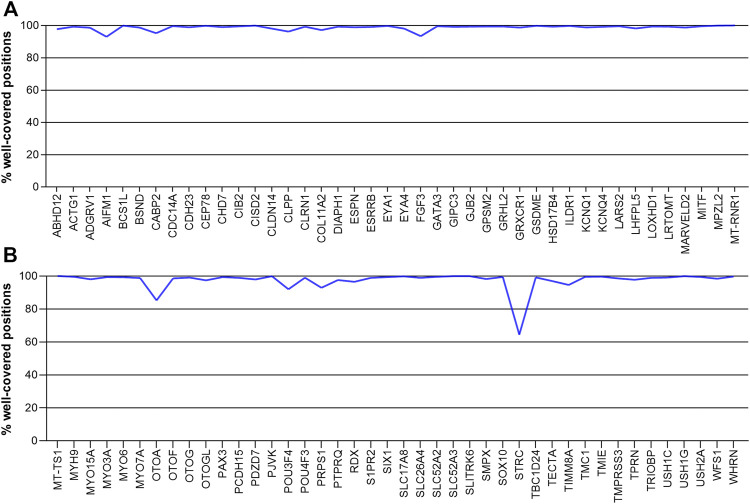
The coverage of deafness-related genes in our sequencing, showing the percentage of gene positions that are well covered. Genes are arranged in alphabetical order. **(A)** *ABHD12*- *MT-RNR1* **(B)** *MT-TS1*-*WHRN*.

Most of the 84 deafness-related genes in our study well covered, with coverage of 90% to nearly 100% of the corresponding gene regions. Only two genes had less than 90% of their regions covered at 20X. They were *OTOA* (85.82%) and *STRC* (64.42%) (Figure 3). These two genes have homologous pseudogenes that are nearly identical to their sequence, explaining the low coverage in our sequencing data.

WGS identified all of the types of known variants associated with hearing loss in the six samples, including single nucleotide variations, insertions/deletions, and copy number variations in the nuclear or mitochondrial genome. More specifically, samples HL-V1, HL-V2 and HL-V3 had heterozygous or homozygous single nucleotide variations and insertions/deletions, while a chromosomal deletion (chr17:g.16670884-20391194, 3.7 Mb) leading to Smith-Magenis syndrome was found in HL-V4. In HL-V5 and HL-V6, homoplasmy and heteroplasmy of the mitochondrial variant m.1555A>G in the *MT-RNR1* gene was detected (Table 1).

**TABLE 1:** The results of whole genome sequencing for six samples with known genetic variation.

Sample ID	Gene	Variant	Consequence	Zygosity	Variant Type	Sequencing Coverage	Ratio
HL-V1	*GJB2*	c.109G>A	Missense	Heterozygous	SNV	40	0.56
	*GJB2*	c.176_191del	Frameshift	Heterozygous	Indel	28	0.44
HL-V2	*SLC26A4*	c.589G>A	Missense	Heterozygous	SNV	20	0.44
	*SLC26A4*	c.919-2A>G	Splicing	Heterozygous	SNV	31	0.49
HL-V3	*MYO7A*	c.3696_3706del	Frameshift	Homozygous	Indel	47	1.00
HL-V4	-	17p11.2 (16670884-20391194) x1 del^#^	-	Heterozygous	CNV	*	0.51
HL-V5	*MT-RNR1*	m.1555A>G	-	Homoplasmy	SNV	4,940	1.00
HL-V6	*MT-RNR1*	m.1555A>G	-	Heteroplasmy	SNV	4,891	0.26

Transcript: GJB2, NM_004004.5; SLC26A4, NM_000441.1; MYO7A, NM_000260.3; MT-RNR1, NC_012920.1. SNV, single nucleotide variant; indel, insertion and deletion; CNV, copy number variant. *, the expected and observed sequence coverage of 17:16670884-20391194 was 31694 and 16235, respectively. #, Smith-Magenis syndrome.

The other three samples (HL-V7, HL-V8 and HL-V9) were known to have CMV infections. The viral load, confirmed by qPCR, differed between the samples. Sequencing reads mapping to CMV reference genome increased substantially with the increase in viral load, with 4,551, 28,924, and 20,876 reads in the samples, respectively (Table 2).

**TABLE 2:** The results of WGS for three samples with known human CMV infection.

Sample ID	CMV Viral Load (Copies/mL by qPCR)	WGS	
		CMV Reads	Human Reads
HL-V7	2.3*10^4	4,551	1,827,820,814
HL-V8	1.5*10^5	28,924	1,799,376,300
HL-V9	1.9*10^6	206,867	1,820,197,488
Negative control	0	0	1,627,205,505

### Pilot Study

The WGS analytic pipeline was pilot tested on 51 de-identified dried blood spots from newborns. Of these, nine (18%) were found to carry pathogenic variants in one of the 84 deafness-related genes (Supplementary Table S3). NM_004004.5:c.109G > A in the *GJB2* gene was detected in five individuals and was the most prevalent in the population. CMV infection was not detected in any of these 51 samples. All pathogenic variants detected by WGS in the pilot study were validated by Sanger sequencing (Supplementary Figure S1).

## Discussion

To facilitate the integration of WGS into newborn screening, we curated and selected 84 deafness-related genes (92 gene-disease pairs) using criteria established by expert groups (Ceyhan-Birsoy et al., 2017; DiStefano et al., 2019). We then validated our WGS analytics pipeline in samples harboring the most common types of deafness-related variants in nuclear and mitochondrial genomes. We revealed that WGS can not only detect these variants, but also detect CMV infection in specimens with different viral loads.

Genetic factors and congenital CMV infection are two major causes of congenital and childhood hearing loss (Shearer et al., 2019). A comprehensive newborn hearing screen that includes physiologic, genetic, and CMV screening would have multiple clinical benefits for newborns (Shearer et al., 2019). Incorporating limited genetic screening into an auditory screening has been widely adopted in large population screens in China (Dai et al., 2019; Wang et al., 2019; Guo et al., 2020). However, CMV screening in newborns has been less well investigated. Recently, Yamamoto and co-authors reported a prospective study of CMV screening in 11900 newborns in Brazil (Yamamoto et al., 2019). These studies demonstrated that either genetic or CMV screening can identify affected newborns who passed the auditory screens. As such, WGS could be a promising supplement to a traditional auditory newborn screening program since it can identify both genetic variants and CMV infections in parallel.

WGS has several advantages for newborn hearing screening. First, limited genetic screens only include a small number of pre-selected variants. This presents an inherent ethnic bias, which is an important drawback in a population with racial and ethnic diversity (Shearer et al., 2019). WGS, however, can analyze the full sequence of selected genes. More importantly, WGS is more powerful than whole-exome sequencing for detecting exome variants (Belkadi et al., 2015). Second, WGS can detect both chromosomal and exon-level CNVs, which are common causes of genetic hearing loss (Shearer et al., 2014). Third, WGS can simultaneously detect genomic variants and CMV infections, as we demonstrated in this study. These features highlight the advantages of WGS over limited genetic screens or WES.

It should be noted that WGS is limited in its ability to detect variants in genes with high homology. Two deafness-related genes (*STRC* and *OTOA*) have homologous regions in the human genome, which explains their low coverage in our WGS data. The *STRC* gene encodes stereocilin, a large extracellular structural protein found in the stereocilia of outer hair cells in the inner ear (Verpy et al., 2001). It is the second most common etiology in cases of mild-to-moderate hearing loss, after the *GJB2* gene (Yokota et al., 2019). *STRC* is highly homologous to pseudogene *pSTRC* (99.6% of coding regions and 98.9% including introns) (Francey et al., 2012). *OTOA* encodes otoancorin, a protein that is important for conditioning proper stimulation of the inner hair cells (Zwaenepoel et al., 2002). It has 99% sequence similarity with exons 22–28 of the pseudogene *OTOAP1*, which generally leads to non-allelic homologous recombination-mediated deletions (Laurent et al., 2021). Considering the high prevalence of these two genes in hearing loss patients and the limitation of WGS, the residual risk should be calculated and the newborns’ guardians should be informed prior to testing.

Allelic heterogeneity, which leads to variable phenotypes, poses challenges to clinical counseling. For example, we identified two cases carrying c.2802T>G (p.Cys934Trp) in the *USH2A* gene (Supplementary Table S3). Although this variant is associated with both Usher syndrome and retinitis pigmentosa, most patients present with nonsyndromic retinitis pigmentosa with normal hearing (Meng et al., 2021; Zhu et al., 2021). To obtain an accurate clinical diagnosis, comprehensively ophthalmic examinations and audiological assessments are required. More importantly, clinical follow-up is needed.

Another challenge of implementing WGS as a screening test in newborns is the burden of variant interpretation (Narravula et al., 2017). To overcome this issue, our group recently developed a semi-automated variant interpretation platform for genetic hearing loss (Peng et al., 2021). This tool semi-automates variant interpretation based on the rules recommended by the American College of Medical Genetics and Genomics and the Association for Molecular Pathology (Peng et al., 2021). It facilitates and standardizes the interpretation of genetic hearing loss variants.

One important caveat that we wish to stress is that the viral load of CMV might be low in peripheral blood specimens. This led to low test sensitivity when dried blood spots from newborns’ heel were used (Boppana et al., 2010). Samples of saliva or umbilical cord blood might be a better alternative. Several studies have demonstrated that saliva specimens are reliable for screening newborns for CMV, offering high sensitivity and specificity (Boppana et al., 2011; Sun and Reichenberger, 2014). Umbilical cord blood, however, has received less investigation. Tagawa and co-authors reported that dried umbilical cord blood samples can be used to identify congenital CMV infections (Tagawa et al., 2009). In addition, Reyes et al. (2020) concluded that dried umbilical cord blood is an alternative to dried blood spots from heels and has better sensitivity.

It is also worth noting that the association between CMV infections and hearing loss relies on the virus loads. Boppana et al. revealed that the risk of hearing loss was low when the viral DNA is less than 5 × 10^3^ pfu/ml in urine or low than 1 × 10^4^ copies/mL in urine (Boppana et al., 2005). In addition, Ross et al. reported that a viral load of <3,500 genomic equivalents per milliliter is associated with a lower risk of hearing loss in children born with asymptomatic congenital infection (Ross et al., 2009). That is, when the CMV infection is revealed by WGS, a quantitative test (i.e., qPCR) is warranted to evaluate the risk.

In summary, WGS can simultaneously detect genetic variants and CMV infections in dried blood spot specimens from newborns. Our study provides proof of principle that genome sequencing can be a promising alternative for newborn screening of hearing loss.

## Data Availability

The original contributions presented in the study are included in the article/Supplementary Material, further inquiries can be directed to the corresponding author. The data reported in this study are also available in the CNGB Nucleotide Sequence Archive (CNSA: https://db.cngb.org/cnsa; accession number CNP0002697).
