# Supplementary material for: Utility of Whole Genome Sequencing for Population Screening of Deafness-Related Genetic Variants and Cytomegalovirus Infection in Newborns
Source: Front Genet. 2022 Apr 29;13:883617. doi: 10.3389/fgene.2022.883617 (PMC9099144; doi:10.3389/fgene.2022.883617)
Supplement: Supplementary file 1 [file DataSheet1.docx]

**Utility of whole genome sequencing for population screening of deafness-related genetic variants and cytomegalovirus infection in newborns**

**Author list：**Jiale Xiang, Hongfu Zhang, Xiangzhong Sun, Junqing Zhang, Zhenpeng Xu, Jun Sun, Zhiyu Peng

**Correspondence:** Zhiyu Peng, pengzhiyu@bgi.com

**Including:** Supplementary Table1-3; Supplementary Figure 1

**Supplementary Table 1** The list of hearing loss related gene-disease pairs

| **Gene** | **Disease association** | **Inheritance** |
| --- | --- | --- |
| *ABHD12* | PHARC syndrome | AR |
| *ACTG1* | Baraitser-winter syndrome 2 | AD |
| *ACTG1* | Nonsyndromic genetic deafness | AD |
| *ADGRV1* | Usher syndrome type 2 | AR |
| *AIFM1* | X-linked hereditary sensory and autonomic neuropathy with deafness | XL |
| *BCS1L* | Bjornstad syndrome | AR |
| *BSND* | Bartter disease type 4a | AR |
| *CABP2* | Nonsyndromic genetic deafness | AR |
| *CDC14A* | Hearing impairment and infertile male syndrome | AR |
| *CDH23* | Usher syndrome type 1 | AR |
| *CDH23* | Nonsyndromic genetic deafness | AR |
| *CEP78* | Cone-rod dystrophy and hearing loss | AR |
| *CHD7* | CHARGE syndrome | AD |
| *CIB2* | Nonsyndromic genetic deafness | AR |
| *CISD2* | Wolfram syndrome | AR |
| *CLDN14* | Nonsyndromic genetic deafness | AR |
| *CLPP* | Perrault syndrome 3 | AR |
| *CLRN1* | Usher syndrome type 3 | AR |
| *COL11A2* | Otospondylomegaepiphyseal dysplasia | AD |
| *COL11A2* | Otospondylomegaepiphyseal dysplasia | AR |
| *DIAPH1* | Diaph1-related sensorineural hearing loss-thrombocytopenia syndrome | AD |
| *ESPN* | Nonsyndromic genetic deafness | AR |
| *ESRRB* | Nonsyndromic genetic deafness | AR |
| *EYA1* | Branchio-oto-renal syndrome | AD |
| *EYA4* | Nonsyndromic genetic deafness | AD |
| *FGF3* | Deafness with labyrinthine aplasia, microtia, and microdontia | AR |
| *GATA3* | Hypoparathyroidism-deafness-renal disease syndrome | AD |
| *GIPC3* | Nonsyndromic genetic deafness | AR |
| *GJB2* | Deafness, autosomal recessive | AR |
| *GJB2* | Syndromic genetic deafness | AD |
| *GPSM2* | Chudley-McCullough syndrome | AR |
| *GRHL2* | Nonsyndromic genetic deafness | AD |
| *GRXCR1* | Nonsyndromic genetic deafness | AR |
| *GSDME* | Autosomal dominant nonsyndromic deafness | AD |
| *HSD17B4* | Perrault syndrome | AR |
| *ILDR1* | Nonsyndromic genetic deafness | AR |
| *KCNQ1* | Jervell and Lange-Nielsen syndrome Obsolete Term | AR |
| *KCNQ4* | Nonsyndromic genetic deafness | AD |
| *LARS2* | Perrault syndrome | AR |
| *LHFPL5* | Nonsyndromic genetic deafness | AR |
| *LOXHD1* | Nonsyndromic genetic deafness | AR |
| *LRTOMT* | Autosomal recessive nonsyndromic deafness 63 | AR |
| *MARVELD2* | Nonsyndromic genetic deafness | AR |
| *MITF* | Waardenburg syndrome type 2 | AD |
| *MPZL2* | Nonsyndromic genetic deafness | AR |
| *MYH9* | Macrothrombocytopenia and granulocyte inclusions with or without nephritis or sensorineural hearing loss | AD |
| *MYO15A* | Nonsyndromic genetic deafness | AR |
| *MYO3A* | Nonsyndromic genetic deafness | AR |
| *MYO6* | Nonsyndromic genetic deafness | AD |
| *MYO7A* | Usher syndrome type 1 | AR |
| *MYO7A* | Nonsyndromic genetic deafness | AD |
| *OTOA* | Nonsyndromic genetic deafness | AR |
| *OTOF* | Autosomal recessive nonsyndromic deafness 9 | AR |
| *OTOG* | Nonsyndromic genetic deafness | AR |
| *OTOGL* | Nonsyndromic genetic deafness | AR |
| *PAX3* | Waardenburg syndrome | AD |
| *PCDH15* | Usher syndrome type 1 | AR |
| *PDZD7* | Deafness, autosomal recessive | AR |
| *PJVK* | Nonsyndromic genetic deafness | AR |
| *POU3F4* | Nonsyndromic genetic deafness | XL |
| *POU4F3* | Nonsyndromic genetic deafness | AD |
| *PRPS1* | PRPS1 deficiency disorder | XL |
| *PTPRQ* | Deafness, autosomal recessive | AR |
| *RDX* | Nonsyndromic genetic deafness | AR |
| *S1PR2* | Nonsyndromic genetic deafness | AR |
| *SIX1* | Branchio-oto-renal syndrome | AD |
| *SLC17A8* | Nonsyndromic genetic deafness | AD |
| *SLC26A4* | Pendred syndrome | AR |
| *SLC52A2* | Brown-Vialetto-van Laere syndrome 2 | AR |
| *SLC52A3* | Brown-Vialetto-van Laere syndrome 1 | AR |
| *SLITRK6* | High myopia-sensorineural deafness syndrome | AR |
| *SMPX* | Nonsyndromic genetic deafness | XL |
| *SOX10* | Waardenburg syndrome type 4C | AD |
| *STRC* | Nonsyndromic genetic deafness | AR |
| *TBC1D24* | DOORS syndrome | AR |
| *TECTA* | Nonsyndromic genetic deafness | AR |
| *TECTA* | Nonsyndromic genetic deafness | AD |
| *TIMM8A* | Deafness dystonia syndrome | XL |
| *TMC1* | Autosomal recessive nonsyndromic deafness 7 | AR |
| *TMC1* | Nonsyndromic genetic deafness | AD |
| *TMIE* | Nonsyndromic genetic deafness | AR |
| *TMPRSS3* | Nonsyndromic genetic deafness | AR |
| *TPRN* | Nonsyndromic genetic deafness | AR |
| *TRIOBP* | Deafness, autosomal recessive | AR |
| *USH1C* | Usher syndrome type 1 | AR |
| *USH1G* | Usher syndrome type 1 | AR |
| *USH2A* | Usher syndrome type 2 | AR |
| *WFS1* | Wolfram-like syndrome | AD |
| *WFS1* | Wolfram syndrome | AR |
| *WHRN* | Usher syndrome type 2D | AR |
| *MT-RNR1* | Mitochondrial nonsyndromic sensorineural deafness with susceptibility to aminoglycoside exposure | MT |
| *MT-TS1* | Mitochondrial nonsyndromic sensorineural deafness | MT |

AR, autosomal recessive; AD, autosomal dominant; XL, X-linked; MT, mitochondrial.

**Supplementary Table 2** The quality of WGS for validation cohort (N=9)

| Item | Median (min-max) |
| --- | --- |
| Raw data(G) | 187.35 (177.94-192.30) |
| Clean data(G) | 185.72 (177.18-191.23) |
| Map data(G) | 176.67 (171.60-181.87) |
| Map rate(%) | 94.95 (93.25-99.71) |
| Depth | 65.22 (61.07-67.46) |
| Rmdup depth | 59.49 (54.4-63.21) |
| Dup rate | 0.09 (0.06-0.11) |
| Coverage >0x(%) | 99.92 (99.90-99.95) |
| Coverage >=4x(%) | 99.83 (99.80-99.89) |
| Coverage >=10x(%) | 99.65 (99.60-99.76) |
| Coverage >=20x(%) | 99.12 (98.94-99.42) |
| Clean fq GC rate(%) | 40.67 (40.52-40.97) |
| Clean fq Q20 rate(%) | 97.76 (97.32-98.54) |
| Clean fq Q30 rate(%) | 91.27 (90.17-95.61) |
| Dedup coverage >=1(%) | 99.91 (99.21-99.94) |
| Dedup coverage >=4(%) | 99.80 (99.07-99.87) |
| Dedup coverage >=10(%) | 99.54 (98.83-99.71) |
| Dedup coverage >=20(%) | 98.47 (97.91-98.86) |
| Dedup coverage >=25(%) | 97.22 (95.91-97.98) |
| Dedup coverage >=30(%) | 95.14 (92.83-96.78) |
| Dedup coverage >=35(%) | 92.30 (88.78-94.07) |
| ChrM dedup coverage >=2000(%) | 98.92 (93.72-99.95) |

**Supplementary Table 3** The results of WGS for nine samples with carried pathogenic variants in deafness-related genes

| Sample ID | Gene | Variant | Zygosity | Coverage | Ratio |
| --- | --- | --- | --- | --- | --- |
| CS-P1 | *GJB2* | c.109G>A, p.Val37Ile | Heterozygous | 38 | 0.49 |
| CS-P2 | *GJB2* | c.109G>A, p.Val37Ile | Heterozygous | 31 | 0.51 |
| CS-P3 | *GJB2* | c.109G>A, p.Val37Ile | Heterozygous | 24 | 0.47 |
| CS-P4 | *GJB2* | c.109G>A, p.Val37Ile | Heterozygous | 38 | 0.51 |
| CS-P5 | *GJB2* | c.109G>A, p.Val37Ile | Heterozygous | 23 | 0.49 |
|  | *SLC26A4* | c.2168A>G, p.His723Arg | Heterozygous | 35 | 0.47 |
| CS-P6 | *USH2A* | c.9815C>T, p.Pro3272Leu | Heterozygous | 29 | 0.62 |
| CS-P7 | *USH2A* | c.2802T>G, p.Cys934Trp | Heterozygous | 29 | 0.43 |
| CS-P8 | *USH2A* | c.2802T>G, p.Cys934Trp | Heterozygous | 31 | 0.53 |
| CS-P9 | *MARVELD2* | c.866G>A, p. p.Trp289* | Heterozygous | 20 | 0.44 |

Transcript: *GJB2*, NM_004004.5; *SLC26A4*, NM_000441.1; *USH2A*, NM_206933.2; *MARVELD2*, NM_001038603

**
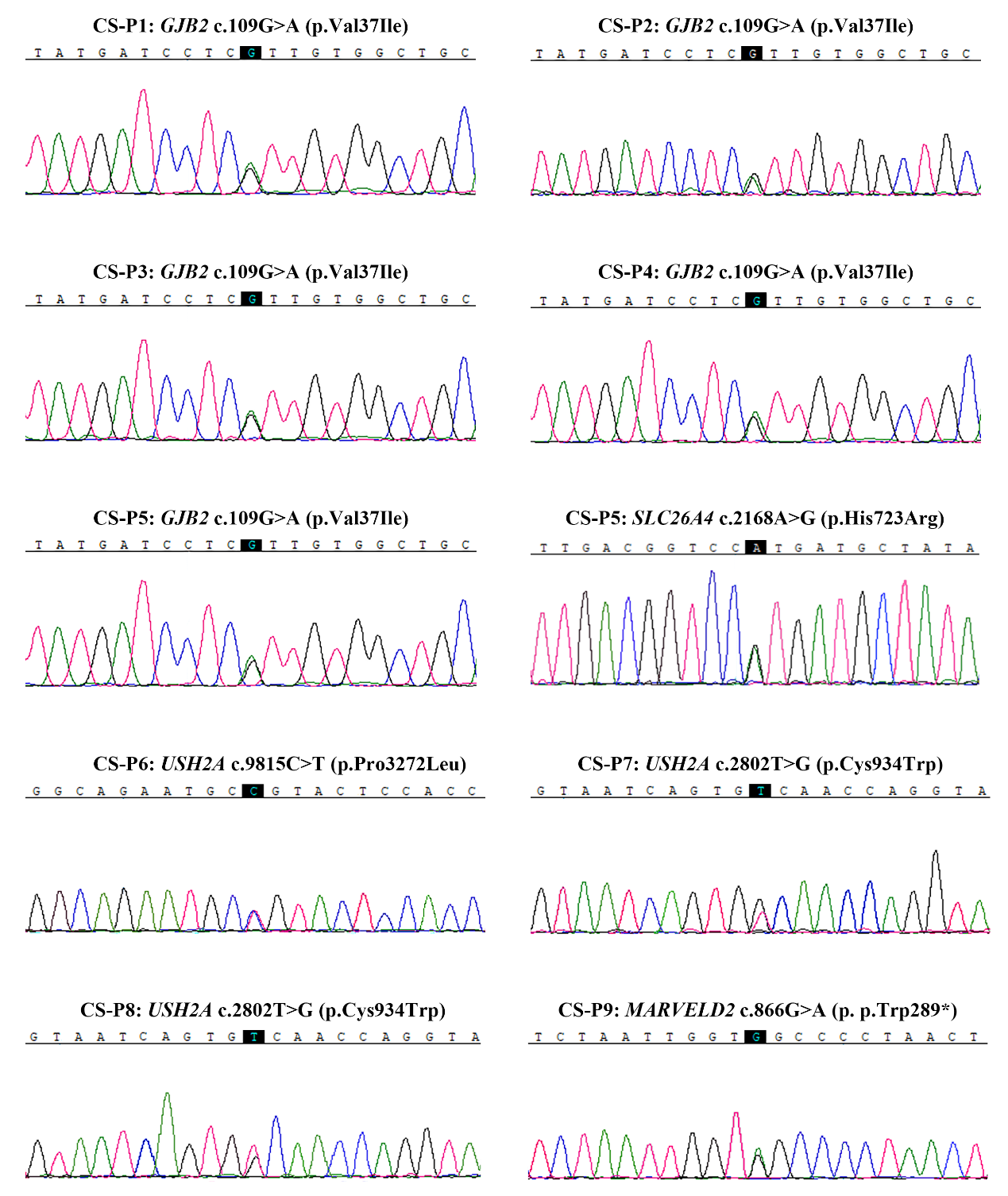
**

**Supplementary Figure 1** The chromatograms of Sanger sequencing of the pathogenic variants detected by WGS in the pilot study.
